# Self-regulation of BAX-induced cell death

**DOI:** 10.18632/oncotarget.11948

**Published:** 2016-09-10

**Authors:** Denis E. Reyna, Evripidis Gavathiotis

**Affiliations:** Department of Biochemistry, Department of Medicine, Albert Einstein Cancer Center, Wilf Family Cardiovascular Research Institute, Albert Einstein College of Medicine, Bronx, NY, USA

**Keywords:** apoptosis, mitochondria, BAX, BCL-2 family, BH3

Apoptosis, a form of programmed cell death, is a process in multicellular organisms responsible for normal tissue development and homeostasis. The intrinsic pathway of apoptosis is principally regulated by protein-protein interactions within the BCL-2 family of proteins, which can prevent or promote mitochondrial dysfunction. There are over twenty BCL-2 family proteins grouped together based on their functional and structural similarities. Specifically, family members are divided into anti-apoptotic and pro-apoptotic proteins and posses up to four BCL-2 Homology (BH) domains, namely BH1, BH2, BH3 and BH4. The BH3 domain is the most conserved and serves as the key domain to mediate protein-protein interactions between the members. Upon a variety of apoptotic stimuli, pro-apoptotic protein BAX triggers the intrinsic apoptotic pathway by inducing mitochondrial outer membrane permeabilization and the release of soluble factors important in caspase activation that are required for apoptosis. The precise mechanisms of BAX activation and inhibition are essential to the understanding of the mitochondrial cell death pathway in physiological and pathological states.

Pro-apoptotic BAX resides primarily in the cytosol in a conformation that requires activation for inducing apoptosis [1]. Interaction of cytosolic BAX with the BH3 domain of BH3-only proteins, such as BIM, induces structural conformational changes on BAX enabling it to translocate to the mitochondria and homo-oligomerize into a deadly membrane pore [2, 3]. Specifically, structural studies have identified that BH3-binding to the N-terminal activation site stimulates conformational changes leading to the release of the C-terminal helix α9 from the hydrophobic groove, which facilitates mitochondrial anchoring and oligomerization of BAX [4, 5]. Therefore, two structural regions critical to BAX activation are available for BAX modulation in the cytosol. Because BAX activation requires the availability of its activators, it is not understood if and how BAX can regulate its activation in the presence of apoptotic insults.

In search of additional mechanisms that keep cytosolic BAX inactive and regulate its activation, we investigated the hypothesis that other proteins may interact with BAX in the cytosol. For several cell lines that are under non-apoptotic stress, we isolated cytosolic fractions from mitochondria using detergent-free solutions. We performed size-exclusion chromatography analysis of the cytosolic fractions and as previously reported, cytosolic BAX eluted as monomers. Surprisingly, we also found that in some cells cytosolic BAX eluted as dimers or both as monomers and dimers [6]. Analysis of other BCL-2 family proteins for potential interactions with BAX excluded the possibility of stable hetero-dimers in the cytosol supporting the concept of cytosolic BAX homo-dimers.

Purification and analysis of recombinant fulllength BAX in dimeric conformation showed that BAX dimers were resistant to the BH3-mediated activation and failed to undergo translocation to the membrane and induce membrane permeabilization [6]. Only when the BAX dimers dissociated into BAX monomers the BAX activation pathway proceeded. Determination of the fulllength crystal structure of BAX revealed that BAX dimers formed an asymmetric conformation with intermolecular interactions involving the N-terminal BAX activation site of one BAX monomer and the C-terminal surface, including C-terminal helix α9, of the other BAX monomer [6]. Because BAX activation by the BH3 binding to the N-terminal activation site induces a conformational change that leads to C-terminal helix α9 release [7], the structural findings suggest that BAX dimer forms an autoinhibited conformation that resists BH3 activation and conformational displacement of helix α9, providing stabilization of the inactive BAX conformation.

**Figure 1:**
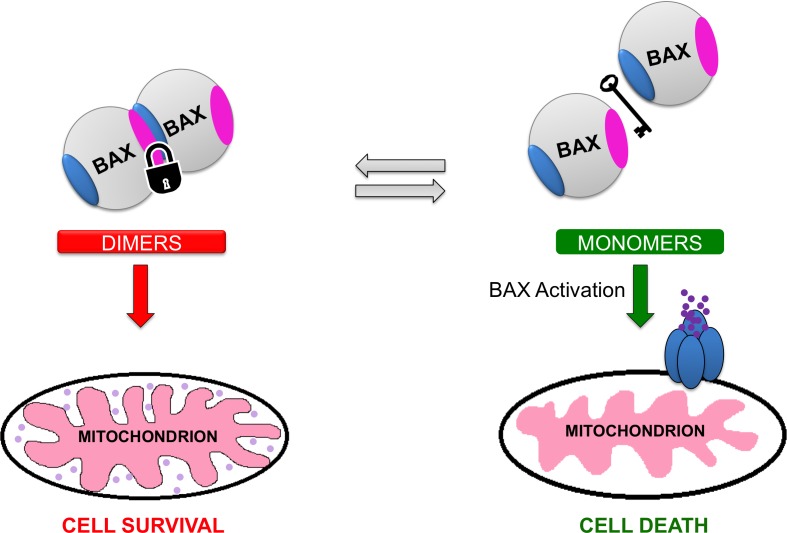
Regulation of cytosolic BAX by autoihibited dimers Cytosolic BAX can adopt a monomeric or a dimeric conformation. The newly characterized cytosolic BAX dimer exhibits an asymmetric conformation in which the N-terminal activation site (blue region) of one BAX monomer interacts with a site at the C-terminal surface (magenta region) of the other BAX monomer. This dimeric conformation suppresses the initiation of structural changes necessary for the activation of BAX. Cellular stressors such as BH3-only proteins can shift the balance from cytosolic BAX dimers to monomers. Eventually, BAX monomers are promptly activated by interaction with the BH3-only proteins promoting the downstream events that lead to cell death.

To interrogate the physiological role of the BAX dimers in apoptosis, we tested mouse embryonic fibroblasts (MEFs) with wild type BAX dimers and dimerization disruptive BAX mutants. Upon BAX expression or stress induction, BAX mutant monomers were readily translocated and oligomerized leading to robust apoptosis induction [6]. On the contrary, BAX dimers were protective to BAX activation and apoptosis induction. Moreover, leukemia cells with monomeric BAX showed faster BAX translocation, oligomerization and apoptosis induction compared to cytosolic BAX dimers in MEFs [6]. Consequently, our data indicate that autoinhibited cytosolic BAX dimers regulate BAX activation and BAX-dependent cell death (Figure 1).

Overall, our new findings support a model in which cytosolic BAX is regulated by the formation of autoinhibited cytosolic BAX dimers that inhibit prompt BAX activation and apoptosis by BH3-only proteins. Structurally, this is achieved in an elegant manner from the interaction of two critical surfaces of the BAX monomer structure that inhibits the essential conformational activation of BAX for membrane translocation. What determines the levels between cytosolic BAX monomers and dimers is still not clear. Post-translational modifications and specific cellular context could be responsible. Moreover, this conformational switch of cytosolic BAX should also affect the active role of BAX in regulating mitochondrial fusion and fission and it should be further investigated. Nevertheless, the discovery of the autoinhited BAX dimer and the structural topology of the interactions provide an insightful understanding of how cytosolic BAX achieves to maintain a reserved conformation that ultimately can regulate the degree of BAX-induced cell death. Beyond this fundamental understanding, the study provides an opportunity for development of pharmacologic modulators of BAX that may engage either the N-terminal site or the C-terminal site, involved in the dimerization, to activate or block BAX activation in diseases of deregulated cell death.
